# Impact of Stunting on Outcomes of Severely Wasted Children (6 Months to 5 Years) Admitted for Inpatient Treatment: A Cross-Sectional Study in an Ethiopian Referral Hospital

**DOI:** 10.3390/children12101294

**Published:** 2025-09-24

**Authors:** Serena Pappalardo, Eleni Hagos Giday, Sisay Zeleke Jijo, Francesco Cavallin, Enzo Facci, Giovanni Putoto, Fabio Manenti, Claudia Banzato, Daniele Trevisanuto, Andrea Pietravalle

**Affiliations:** 1 Doctors with Africa CUAMM, Wolisso P.O. Box 250, Ethiopia; serena.pappalardo@aulss6.veneto.it (S.P.); e.hagos@cuamm.org (E.H.G.); e.facci@cuamm.org (E.F.); 2 “St. Luke Catholic Hospital and College of Nursing & Midwifery”, Wolisso P.O. Box 250, Ethiopia; sisay8582@gmail.com; 3 Independent Statistician, 36020 Solagna, Italy; 4 Doctors with Africa CUAMM, 35121 Padua, Italy; g.putoto@cuamm.org (G.P.); f.manenti@cuamm.org (F.M.); 5 Department of Pediatrics, Woman’s and Child’s University Hospital of Verona, 37126 Verona, Italy; claudia.banzato@aovr.veneto.it; 6 Department of Women’s and Children’s Health, University Hospital of Padova, 35128 Padova, Italy; daniele.trevisanuto@unipd.it

**Keywords:** malnutrition, low-income countries, wasting, stunting, treatment outcomes

## Abstract

**Background**: Undernutrition is a major public health concern, accounting for nearly half of global under-five mortalities and leading to serious long-term consequences for those who survive. Most nutritional screening programs give priority to acute undernutrition (wasting). The co-presence of chronic undernutrition (stunting) has been shown to have the highest risk of mortality. To date, few studies have assessed outpatient treatment outcomes of children with wasting + stunting (WaSt), with some inconsistencies in results and only one study having investigated the outcome of patients requiring hospitalization. The aim of the present study is to investigate the impact of stunting on the outcomes of severely wasted children admitted for inpatient treatment in an Ethiopian referral hospital. **Methods**: A retrospective cross-sectional study was conducted to compare treatment outcomes (length of hospital stay, weight gain, recovery rate, readmission rate) of wasted and WaSt children admitted to “St. Luke Catholic Hospital and College of Nursing and Midwifery” between January 2018 and February 2023. **Results**: The analysis comprised 616 children aged 6–60 months and stunting was diagnosed in 559 children (90.7%). Children with stunting had a longer length of stay (median difference 3 days, 95% confidence interval 0 to 5; *p* = 0.03) and improved weight gain (median difference 4 g/kg/day, 95% confidence interval 0 to 4; *p* = 0.002) compared to children without stunting. Discharge rate (*p* = 0.99) and readmission rate (*p* = 0.25) were not statistically different between children with or without stunting. **Conclusions**: Stunting was found to be present in most children admitted for severe wasting to the Stabilization Centers in a sub-Saharan setting. Stunting was associated with longer hospitalization and greater weight gain, but discharge and readmission rate were comparable between children with or without stunting.

## 1. Introduction

Undernutrition represents a major public health concern, being the underlying cause of almost half of global child mortalities. The long-term impacts of childhood undernutrition include lower educational achievement, lower economic productivity, and increased risk of non-communicable disease [1].

Recent estimates suggest that 148 million children under 5 years are affected by chronic undernutrition (stunting) and 48 million by the acute form (wasting) [2], while 15.9 million suffer from both concurrently, with an estimated prevalence of 3% (ranging from 0 to 8%) [3]. In Ethiopia, the prevalence of WaSt has been estimated to be between 4.82% and 5.8%, with a marked decreasing trend in the last 20 years [4,5,6].

Most nutritional screening programs prioritize the identification of wasting as a marker of acute undernutrition associated with the greatest risk of morbidity and death. However, stunting and wasting share common risk factors, and are both associated with a much higher risk of death, with wasting showing roughly twice the risk of stunting and concurrent wasting and stunting (WaSt) having the highest risk of all [7,8]. Wasting and stunting are already present at birth in a significant proportion of children, and this can lead to further growth failure in infancy and childhood [8]. Concurrent wasting and stunting are more prevalent in boys than girls [3,9,10,11], with peaks in ages between 12 and 30 months [3,11,12,13,14,15]. Experiencing repeated episodes of wasting can increase the likelihood of developing stunting [14,16,17,18,19]. To a lesser extent, stunting can also influence the onset of wasting [12,17]. The coexistence of the two conditions therefore leads to an accumulation of vulnerabilities throughout an individual’s life. This underscores the need for cohesive policies and the implementation of services and activities to prevent both wasting and stunting [8].

The management of acute malnutrition involves the “Community Management of Acute Malnutrition (CMAM) program” [20]. The CMAM program identifies malnourished children at community level and refers them to Stabilization Centers (SCs) or Outpatient Treatment Programs (OTPs) according to the presence or absence of complications. Malnourished children without medical complications are treated in OTPs, which provide routine medical treatment and nutritional rehabilitation. Those with medical complications are admitted to SC until their clinical conditions are stabilized and complications resolved.

Stunting is highly prevalent among wasted children admitted to OTPs, with WaSt ranging from 48.7% to 82.2% [11,13,16]. To date, only a few studies have assessed outpatient treatment outcomes of children with WaSt and have reported heterogeneous findings [8]. Moreover, only one study has investigated the outcome of children with WaSt requiring hospitalization in an SC [21].

Given the lack of data in the literature, the present study aimed to assess whether stunting might be associated with impaired treatment outcomes (such as length of hospitalization, weight gain, discharge rate, mortality rate, and readmission rate) in the most vulnerable category of severely wasted children who were admitted for inpatient treatment in an Ethiopian referral hospital.

## 2. Materials and Methods

### 2.1. Study Design

This retrospective cross-sectional study compared treatment outcomes in severely wasted children with or without stunting who were admitted to the nutritional rehabilitation unit of the St. Luke Wolisso Hospital (Wolisso, Ethiopia) between January 2018 and February 2023. The reporting followed the EQUATOR STROBE checklist (Supplementary Materials) [22].

### 2.2. Setting

The “St. Luke Catholic Hospital and College of Nursing and Midwifery” is located in Wolisso (Ethiopia) and is the referral hospital in Oromiya region that has a population of about 1.1 million inhabitants. As part of a network of healthcare facilities involved in the national nutrition program, the “St. Luke” Hospital acts as a Stabilization Center for the inpatient care of undernourished children with complications, as well as an Outpatient Treatment Unit for the rehabilitation phase after discharge [23].

### 2.3. Patients

#### 2.3.1. Inclusion Criteria

All children aged 6–60 months who were admitted for severe wasting between January 2018 and February 2023 were included.

#### 2.3.2. Exclusion Criteria

Children with major malformations, HIV, tuberculosis, and/or cerebral palsy were excluded.

### 2.4. Definitions

The St. Luke’s Hospital management protocol for children with severe acute malnutrition follows WHO guidelines [24]. Severe wasting is defined as weight/height < −3 standard deviations [24]. Stunting is classified as moderate stunting (height/age ≥ −3 and < −2 standard deviations) or severe stunting (height/age < −3 standard deviations) [24]. Edema is identified as a bilateral pitting swelling due to build-up of fluid in the body’s tissues, which starts in the feet (+) and can progress up to the legs (++) and the rest of the body, including the face (+++) [24]. The clinical presentation of wasting includes kwashiorkor (characterized by edema as a distinctive feature, dermatoses, hypopigmented dry, sparse and brittle hair, distended abdomen, and hepatomegaly), marasma (characterized by muscle wasting, xerotic, wrinkled and loose skin due to the loss of subcutaneous fat) and intermediate states of marasmic kwashiorkor [25].

WHO guidelines state that infants and children 6–59 months old with severe wasting and/or nutritional edema who have any of the following characteristics should be referred and admitted for inpatient care: one or more Integrated Management of Childhood Illness (IMCI) danger signs (convulsions, inability to drink or breastfeed, vomiting everything, and being lethargic or unconscious); acute medical problems; severe nutritional edema (+++); and poor appetite (failed the appetite test) [24].

Infants and children aged 6–59 months with severe wasting and/or nutritional edema who are admitted to inpatient care can be discharged and transferred to outpatient care when they do not have any danger signs for at least 24–48 h prior to transfer time; the medical problems that prompted their admission have resolved to the extent there is no longer requirement for inpatient care; they do not have ongoing weight loss (among children admitted with wasting only who did not have nutritional edema at any time); their nutritional edema is no longer grade +++ and is resolving; they have a good appetite; and when all attempts have been made to refer children with medical problems needing mid- or long-term follow-up care and with a significant association with nutritional status to appropriate care/support services and/or the limits of inpatient care have been reached [24].

If any of the following characteristics appear after discharge, the patient should be referred and readmitted for hospital care: one or more Integrated Management of Childhood Illness (IMCI) danger signs (convulsions, inability to drink or breastfeed, vomiting everything, and being lethargic or unconscious); acute medical problems; severe nutritional edema (+++); poor appetite (failed the appetite test) [24].

### 2.5. Data Collection

All data were retrospectively and anonymously retrieved from the hospital records. Data collection included child demographics (age and sex), nutritional status (wasting and stunting), relevant comorbidities (sepsis, anemia, pneumonia, malaria), and information about outcome measures (length of hospital stay, weight gain, discharge, readmission).

### 2.6. Outcome Measures

The outcome measures included the length of hospital stay (days), the weight gain during hospitalization (g/kg/die), the discharge rate and the readmission rate.

### 2.7. Statistical Analysis

Categorical data were summarized as frequency and percentage, and numerical data as mean and standard deviation (SD). Child characteristics were compared between stunted and non-stunted children using Student’s *t* test, the chi squared test, and Fisher’s exact test. The association between length of hospital stay and weight gain with age and sex was explored using two linear regression models. The primary analysis compared the outcome measures between stunted and non-stunted children; the unadjusted analysis employed the chi squared test and Fisher’s exact test (categorical data), or Student’s *t* test (numerical data); and the adjusted analyses employed linear and logistic regression models including stunting and a set of clinically relevant confounders (age, sex, edema and comorbidity status). The logistic regression model of readmission rate was fitted using Firth’s bias reduction method [26]. As additional sub-analysis, the outcome measures were stratified according to wasting and stunting severity with descriptive purpose thus no statistical comparisons were performed. The secondary analysis investigated the association between the outcome measures and stunting (height/age) and wasting (weight/height) as continuous variables, the unadjusted analysis employed linear and logistic regression models including height/age and weight/height as linear and quadratic terms, and the adjusted analyses employed linear and logistic regression models including height/age and weight/height as linear and quadratic terms and a set of clinically relevant confounders (age, sex, edema and comorbidity status). Effect sizes were reported for stunting, height/age, and weight/height as mean difference (MD) or odds ratio (OR), with a 95% confidence interval (CI). Missing data were limited to weight gain for a few subjects; hence, complete case analyses were performed. All tests were 2-sided and a *p* < 0.05 was considered statistically significant. Statistical analysis was carried out using R 4.4 (R Foundation for Statistical Computing, Vienna, Austria) [27].

## 3. Results

### 3.1. Patient Characteristics

Overall, the analysis comprised 616 children aged 6–60 months who were admitted for severe wasting during the study period. Child characteristics at admission are summarized in Table 1. Stunting was diagnosed in 559 children (90.7%), including 52 with moderate stunting and 507 with severe stunting, while the remaining 57 children (9.3%) had severe wasting without stunting at admission. At admission, children with stunting were older (mean 21.6 vs. 18.3 months, *p* = 0.004) and less likely to present with edema (10.7% vs. 24.5%, *p* = 0.004) or kwashiorkor (10.7% vs. 28.1%, *p* < 0.001) (Supplementary Table S1). The mean length of hospital stay was 16.9 days (SD 10.8) and the mean weight gain was 12.4 g/kg/day (SD 13.1) (Table 1). Child age was not associated with hospital length of stay (*p* = 0.19) or weight gain (*p* = 0.38). Child gender was not associated with hospital length of stay (*p* = 0.54) or weight gain (*p* = 0.95). Overall, 558 children (90.6%) were discharged, 14 (2.3%) were transferred to another center, 17 (2.7%) abandoned treatment, 3 (0.5%) did not respond to treatment, and 24 (3.9%) died. The readmission rate was 22/592 (3.7%) (Table 1).

### 3.2. Primary Analysis: Comparison of the Outcome Measures Between Children with or Without Stunting

The comparison of the outcome measures between children with or without stunting is displayed in Table 2. Children with stunting had a longer length of stay (MD 2.7 days, 95% CI 0.1 to 5.2) and improved weight gain (MD 5.2 g/kg/day, 95% CI 1.0 to 9.4) compared to children without stunting. However, the discharge (OR 1.02, 95% CI 0.35 to 2.96) and readmission rates (OR 4.90, 95% CI 0.28 to 81.84) were not statistically different between children with or without stunting. These findings were confirmed when adjusting for clinically relevant confounders (Table 2).

Table 3 displays the outcome measures within specific strata according to wasting and stunting severity. This summary has only a descriptive purpose thus no statistical comparisons were performed.

### 3.3. Secondary Analysis: Association Between the Outcome Measures and Stunting and Wasting as Continuous Variables

The association between the outcome measures and stunting (height/age) and wasting (weight/height) as continuous variables is displayed in Table 4. A longer length of stay was associated with lower height/age (MD −0.4 days, 95% CI −0.7 to −0.1) and weight/height (MD −1.3 days, 95% CI −2.1 to −0.4). Improved weight gain was associated with lower height/age (MD −1.0 g/kg/day, 95% CI −1.4 to −0.6) and higher weight/height as both linear term (MD 6.2 g/kg/day, 95% CI 1.1 to 11.3) and quadratic term (MD 0.8 g/kg/day, 95% CI 0.3 to 1.3). Discharge rate was associated with higher weight/height (OR 1.34, 95% CI 1.05 to 1.70) but not with height/age (OR 1.02, 95% CI 0.90 to 1.15). Readmission rate was associated with lower height/age (OR 0.77, 95% CI 0.64 to 0.94) but not with weight/height (OR 1.26, 0.82 to 2.10). These findings were confirmed when adjusting for clinically relevant confounders (Table 4).

**Figure 1:**
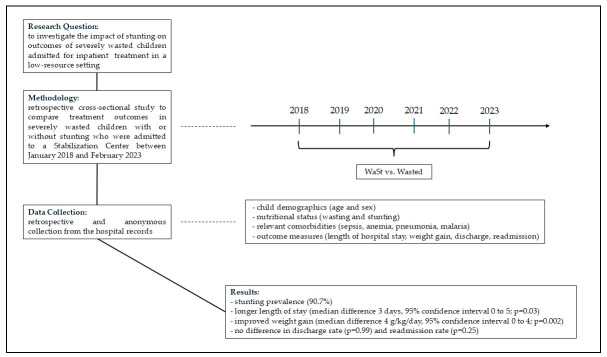
Descriptive flow of the study.

Figure 1 displays a descriptive flow of the study to improve the readability.

## 4. Discussion

Our findings highlighted that stunting involved around nine out of ten children among those who were admitted to the nutritional rehabilitation unit for severe wasting. It is worth noting that children hospitalized with WaSt had a longer duration of hospitalization and a greater weight gain compared to those with only wasting, while discharge and readmission rates were comparable between children with or without stunting.

For some time, stunting and wasting have been considered as distinct conditions of malnutrition, but the cumulative evidence in the last decade has improved the understanding of their relationship [8]. The current literature suggests that stunting and wasting have common causal pathways, often co-occur in the same child, and that concurrent stunting/wasting impairs child prognosis [8]. Moreover, multiple episodes of wasting may increase the risk of stunting, while the severity of stunting may increase the risk of wasting [8].

Overall, we found a high prevalence of stunting among children who were admitted to the nutritional rehabilitation unit for severe wasting. Our prevalence was higher than the figures reported by a similar study on WaSt children requiring hospitalization in SC (60%) and by previous studies on WaSt children treated in OTPs (48.7–82.2%) [8,9,10,11]. Data from the literature on WaSt children treated in OTPs has already informed about the widespread occurrence of concurrent stunting/wasting among malnourished children. As concurrent stunting/wasting impairs child prognosis [8], we can expect that a larger portion of WaSt children might need hospitalization, and our figures suggest that such a portion may be up to 90%.

In our series, the co-occurrence of stunting and wasting suggested some implications on prolonged length of hospital stay, which may be reasonable given the compromised clinical situation of such patients. In the only previous study analyzing the prognostic role of stunting compresence in wasted children during hospitalization, the authors did not find any statistically significant impact of stunting on hospital length of stay [21]. Although the detected mean difference of three days may seem clinically insignificant, it should be considered how even just a few days can negatively impact compliance, resulting in hospital self-discharge and treatment abandonment. In fact, in low-resource settings, prolonged hospitalizations have an impact that is, in most cases, unsustainable, both economically and in terms of family management. Nonetheless, treatment abandonment was comparable among wasting children with or without stunting in our series.

Another interesting aspect of treatment for child malnutrition is growth velocity during hospitalization, which was higher in our WaSt children. This finding was in agreement with previous studies on WaSt children who were treated in SC [21] and OPTs [28]. Conversely, other data from Niger on patients treated in OPTs found no difference in weight gain [29]. In the present study, this difference in growth, which occurs starting from similar values of weight/height ratio and no difference in the presence of edema in the two groups, could suggest a role of stunting in determining an increased catch-up growth. However, also given the inconsistency of results found in studies on OTPs patients, it remains difficult to draw definitive conclusions. The co-occurrence of stunting and wasting has sometimes been associated with impaired treatment outcomes. Among children treated in OTPs, Odei Obeng-Amoako et al. reported higher non-response rate and lower recovery rate in the case of WaSt [29], while Isanaka et al. found that response to treatment for severe acute wasting was not influenced by stunting occurrence [28]. In the study conducted by Tripoli et al. on children admitted for inpatient treatment, the presence of associated pathologies and mortality were much higher in the stunting group [21]. In our series, the discharge rate was comparable for children with or without stunting. However, the literature offers conflicting results from other studies [28,29] and higher mortality risk in children with WaSt [7,8,21], which suggest caution in evaluating the response to treatment. It is noteworthy that the low readmission rate might have been influenced by local barriers (such as time or transport facilities) which prevented the parents from taking the child back to the SC in cases where conditions worsened.

Our study contributes to the literature on child malnutrition, offering further information on the prognostic role of stunting on the outcomes of the most vulnerable category of severely wasted children admitted to hospital in low-resource settings.

The finding of an association with unfavorable inpatient outcomes can provide a further element of advocacy about the need for a most appropriate way to identify the risk level of these patients, as already underlined in recent studies [30,31], and define specific care paths that involve the screening phase as well as out- and inpatient treatment. To address maternal and child malnutrition, direct and indirect health and non -healthcare sector interventions, alongside crosscutting strategies for nutrition support and integration, are necessary [32]. Achieving this goal may require the use of Clinical Networks [33] and Standardized Care Pathways [34]. The last technical briefing paper from the Wasting and Stunting Technical Interest Group (WaSt TIG) suggests specific guidance for clinical practice. Public health prevention strategies should target both wasting and stunting drivers, including those acting before birth; interventions should seek to improve adolescent girls’ and women’s health and nutrition, focusing on pregnant women’s nutritional status as well as that of mothers of at-risk infants; targeting for prevention programs should prioritize multiple risk factors for undernutrition rather than anthropometric cut off or geographical targeting; mechanisms to identify and target WaSt children with appropriate treatment should be prioritized and scaled-up within wasting treatment programs; and younger children (<24 months) should be prioritized for treatment interventions, given their increased risk of concurrent wasting and stunting, adopting strategies to better identify sever wasting and WaSt occurrence to maximize coverage of treatment. The “Management of at Risk Mothers and Infant Care Pathway” can be used as a strategy for scaling-up interventions for infants aged 0–6 months [35].

However, this study also has some limitations that should be considered. Firstly, the single-center design limits the generalizability of the findings to similar settings. Secondly, the retrospective design precludes any causal associations between stunting and treatment outcomes. In addition, the unbalanced size of the two groups (stunted and non-stunted children) suggests caution in the interpretation of the findings. Thirdly, some interesting data (such as long-term assessments and follow-up information) were unfortunately unavailable due to the impossibility of accessing the registers present in the peripheral health centers where follow-up and post-discharge treatment are carried out. The absence of these data limits our ability to have a complete picture of mortality and relapse.

## 5. Conclusions

Stunting was found to be present in most children admitted for severe wasting to the nutritional rehabilitation unit in a sub-Saharan setting. Stunting was associated with longer hospitalization and greater weight gain, but discharge and readmission rate were comparable between children with or without stunting. The present study provides an insight into the more fragile category of WaSt children who require hospitalization, emphasizing the need for advocacy regarding the WaSt condition. Further studies are needed to confirm and consolidate our findings.

## Figures and Tables

**Table 1:** Characteristics of children aged 6–60 months who were admitted for severe wasting.

Characteristics at Admission	All Patients (n = 616)
Females	253 (41.1%)
Males	363 (58.9%)
Age at admission, months:	
All children	21.3 (11.3)
Females	20.8 (10.5)
Males	21.7 (11.8)
Nutritional status at admission:	
Severe wasting (weight/height < −3 SD)	57 (9.3%)
Severe wasting and moderate stunting (−3 SD ≤ height/age < −2 SD)	52 (8.4%)
Severe wasting and severe stunting (height/age < −3 SD)	507 (82.3%)
Weight/height, SD	−4.3 (1.0)
Height/age, SD	−4.9 (2.4)
Edema:	
No	542 (88.0%)
+	11 (1.8%)
++	37 (6.0%)
+++	26 (4.2%)
Wasting clinical presentation:	
Kwashiorkor	34 (5.5%)
Marasma	540 (87.7%)
Kwashiorkor and marasma	42 (6.8%)
Sepsis	28 (4.5%)
Anemia	19 (3.1%)
Pneumonia	31 (5.0%)
Malaria	Nil
Length of hospital stay, days	16.9 (10.8)
Weight gain, g/kg/day	12.4 (13.1)
Discharge status:	
Discharged	558 (90.6%)
Referred to another center	14 (2.3%)
Abandoned treatment	17 (2.7%)
No response to treatment	3 (0.5%)
Dead	24 (3.9%)
Readmissions among live patients	22/592 (3.7%)

Data summarized as n (%) or mean (SD). SD: standard deviation. Data on weight gain were not available in four children.

**Table 2:** Outcome measures in children admitted for severe wasting with or without stunting.

Outcome Measure	Children with Severe Wasting and Stunting (n = 559)	Children with Severe Wasting Without Stunting (n = 57)	Unadjusted Analysis		Adjusted Analysis	
			MD or OR (95% CI)	*p*-Value	MD or OR (95% CI)	*p*-Value
Length of hospital stay, days	17.2 (10.9)	14.5 (9.0)	2.7 (1.0 to 5.2)	0.04	3.0 (0.1 to 5.9)	0.04
Weight gain, g/kg/day	12.9 (12.8)	7.7 (15.3)	5.2 (1.0 to 9.4)	0.02	4.2 (0.6 to 7.8)	0.02
Discharge ^a^	508/548 (92.7%)	50/54 (92.6%)	1.02 (0.35 to 2.96)	0.99	0.98 (0.28 to 2.59)	0.98
Readmission ^b^	22/536 (4.1%)	0/56 (0.0%)	4.90 (0.28 to 81.84)	0.25	4.83 (0.65 to 617.85)	0.15

Data summarized as n (%) or mean (SD). CI: confidence interval. MD: mean difference. OR: odds ratio. SD: standard deviation. Data on weight gain were not available in four children. ^a^ Transferred patients were not included in this comparison. ^b^ Dead patients were not included in this comparison.

**Table 3:** Outcome measures within specific strata according to stunting severity.

Outcome Measure	Children with Severe Wasting and Moderate Stunting (n = 52)	Children with Severe Wasting and Severe Stunting (n = 507)	Children with Severe Wasting Without Stunting (n = 57)
Length of stay, days	17.4 (10.3)	17.2 (11.0)	14.5 (9.0)
Weight gain, g/kg/day	8.4 (7.4)	13.4 (13.2)	7.7 (15.3)
Discharge ^a^	47/50 (94.0%)	461/498 (92.6%)	50/54 (92.6%)
Readmission ^b^	2/50 (4.0%)	20/486 (4.1%)	0/56 (0.0%)

Data summarized as n (%) or mean (SD). SD: standard deviation. Data on weight gain were not available in four children ^a^ Transferred patients were not included in this summary. ^b^ Dead patients were not included in this summary.

**Table 4:** Outcome measures and stunting and wasting as continuous variables.

Outcome Measure	Stunting (Height/Age) and Wasting (Weight/Height)	Unadjusted Analysis		Adjusted Analysis	
		MD or OR (95% CI)	*p*-Value	MD or OR (95% CI)	*p*-Value
Length of hospital stay, days	Height/age	−0.4 (−0.7 to −0.1)	0.02	−0.5 (−0.9 to −0.2)	0.003
	Weight/height	−1.3 (−2.1 to −0.4)	0.002	−1.5 (−2.3 to −0.7)	<0.001
Weight gain, g/kg/day	Height/age	−1.0 (−1.4 to −0.6)	<0.001	−0.8 (−1.2 to −0.4)	<0.001
	Weight/height	6.2 (1.1 to 11.3)	0.02	6.8 (1.7 to 11.8)	0.008
	[Weight/height]^2^	0.8 (0.3 to 1.3)	0.001	0.8 (0.3 to 1.3)	<0.001
Discharge ^a^	Height/age	1.02 (0.90 to 1.15)	0.79	1.03 (0.91 to 1.17)	0.68
	Weight/height	1.34 (1.05 to 1.70)	0.01	1.34 (1.04 to 1.69)	0.02
Readmission ^b^	Height/age	0.77 (0.64 to 0.94)	0.009	0.78 (0.64 to 0.94)	0.01
	Weight/height	1.26 (0.82 to 2.10)	0.33	1.27 (0.83 to 2.12)	0.32

Data summarized as n (%) or mean (SD). SD: standard deviation. CI: confidence interval. MD: mean difference. OR: odds ratio. Data on weight gain were not available in four children. ^a^ Transferred patients were not included in this comparison. ^b^ Dead patients were not included in this comparison.

## Data Availability

The original contributions presented in the study are included in the article/Supplementary Materials, further inquiries can be directed to the corresponding author.

## Supplementary Materials

The following supporting information can be downloaded at: https://www.mdpi.com/article/10.3390/children12101294/s1, STROBE Statement—Checklist of items that should be included in reports of cross-sectional studies; Table S1: Characteristics of children aged 6–60 months who were admitted for severe wasting with or without stunting.

## Abbreviations

WaSt	Concurrency of Wasting and Stunting
CMAM program	Community Management of Acute Malnutrition program
OTPs	Outpatient Treatment Programs
SCs	Stabilization Centers
