# Supplementary material for: Impact of Stunting on Outcomes of Severely Wasted Children (6 Months to 5 Years) Admitted for Inpatient Treatment: A Cross-Sectional Study in an Ethiopian Referral Hospital
Source: Children (Basel). 2025 Sep 24;12(10):1294. doi: 10.3390/children12101294 (PMC12562514; doi:10.3390/children12101294)
Supplement: Supplementary file 1 [file children-12-01294-s001.zip › children-3780373-supplementary.pdf]

**SUPPLEMENTARY MATERIAL**

**STROBE Statement—Checklist of items that should be included in reports of *cross-sectional studies***

| | Item No | Recommendation | Page No |
|---|---|---|---|
| **Title and abstract** | 1 | (*a*) Indicate the study's design with a commonly used term in the title or the abstract | Page 1 |
| | | (*b*) Provide in the abstract an informative and balanced summary of what was done and what was found | Page 1 |
| **Introduction** | | | |
| Background/rationale | 2 | Explain the scientific background and rationale for the investigation being reported | Page 2 |
| Objectives | 3 | State specific objectives, including any prespecified hypotheses | Page 2 |
| **Methods** | | | |
| Study design | 4 | Present key elements of study design early in the paper | Page 2-3 |
| Setting | 5 | Describe the setting, locations, and relevant dates, including periods of recruitment, exposure, follow-up, and data collection | Page 2 |
| Participants | 6 | (*a*) Give the eligibility criteria, and the sources and methods of selection of participants | Page 3 |
| Variables | 7 | Clearly define all outcomes, exposures, predictors, potential confounders, and effect modifiers. Give diagnostic criteria, if applicable | Page 3 |
| Data sources/ measurement | 8 | For each variable of interest, give sources of data and details of methods of assessment (measurement). Describe comparability of assessment methods if there is more than one group | Page 3-4 |
| Bias | 9 | Describe any efforts to address potential sources of bias | N/A |
| Study size | 10 | Explain how the study size was arrived at | Page 4 |
| Quantitative variables | 11 | Explain how quantitative variables were handled in the analyses. If applicable, describe which groupings were chosen and why | Pages 4 |
| Statistical methods | 12 | (*a*) Describe all statistical methods, including those used to control for confounding | Pages 4 |
| | | (*b*) Describe any methods used to examine subgroups and interactions | Pages 4 |

| | | | |
|---|---|---|---|
| | | (*c*) Explain how missing data were addressed | Page 4 |
| | | (*d*) If applicable, describe analytical methods taking account of sampling strategy | N/A |
| | | (*e*) Describe any sensitivity analyses | Pages 4 |
| **Results** | | | |
| Participants | 13 | (a) Report numbers of individuals at each stage of study—eg numbers potentially eligible, examined for eligibility, confirmed eligible, included in the study, completing follow-up, and analysed | Page 4 |
| | | (b) Give reasons for non-participation at each stage | N/A |
| | | (c) Consider use of a flow diagram | N/A |
| Descriptive data | 14 | (a) Give characteristics of study participants (eg demographic, clinical, social) and information on exposures and potential confounders | Page 4 and Table 1 |
| | | (b) Indicate number of participants with missing data for each variable of interest | Table 1 |
| Outcome data | 15 | Report numbers of outcome events or summary measures | Page 5 and Table 2 |
| Main results | 16 | (*a*) Give unadjusted estimates and, if applicable, confounder-adjusted estimates and their precision (eg, 95% confidence interval). Make clear which confounders were adjusted for and why they were included | Page 5 and Table 2 |
| | | (*b*) Report category boundaries when continuous variables were categorized | N/A |
| | | (*c*) If relevant, consider translating estimates of relative risk into absolute risk for a meaningful time period | N/A |
| Other analyses | 17 | Report other analyses done—eg analyses of subgroups and interactions, and sensitivity analyses | Page 6 and Tables 3-4 |
| **Discussion** | | | |
| Key results | 18 | Summarise key results with reference to study objectives | Page 7 |
| Limitations | 19 | Discuss limitations of the study, taking into account sources of potential bias or imprecision. Discuss both direction and magnitude of any potential bias | Page 8 |

| | | | |
|---|---|---|---|
| Interpretation | 20 | Give a cautious overall interpretation of results considering objectives, limitations, multiplicity of analyses, results from similar studies, and other relevant evidence | Pages 7-8 |
| Generalisability | 21 | Discuss the generalisability (external validity) of the study results | Pages 7-8 |
| **Other information** | | | |
| Funding | 22 | Give the source of funding and the role of the funders for the present study and, if applicable, for the original study on which the present article is based | Page 8 |

**Supplementary Table S1. Characteristics of children aged 6-60 months who were admitted for severe wasting with or without stunting**

| Characteristics at admission | Children with severe wasting and stunting (n=559) | Children with severe wasting without stunting (n=57) | p-value |
|---|---|---|---|
| Females<br>Males | 225 (40.3%)<br>334 (59.7%) | 28 (49.1%)<br>29 (50.9%) | 0.25 |
| Age at admission, months | 21.6 (11.3) | 18.3 (10.7) | 0.03 |
| Weight/height, SD | -4.3 (1.0) | -4.4 (1.3) | 0.49 |
| Height/age, SD | -5.4 (1.9) | -0.3 (1.5) | <0.001 |
| Edema:<br>No<br>+<br>++<br>+++ | <br>499 (89.3%)<br>11 (1.9%)<br>29 (5.2%)<br>20 (3.6%) | <br>43 (75.5%)<br>9 (0.0%)<br>8 (14.0%)<br>6 (10.5%) | 0.004 |
| Wasting clinical presentation:<br>Kwashiorkor<br>Marasma<br>Kwashiorkor and marasma | <br>23 (4.1%)<br>499 (89.3%)<br>37 (6.6%) | <br>11 (19.3%)<br>41 (71.9%)<br>5 (8.8%) | <0.001 |
| Sepsis | 27 (4.8%) | 1 (1.8%) | 0.50 |
| Anemia | 16 (2.9%) | 3 (5.3%) | 0.41 |
| Pneumonia | 27 (4.8%) | 4 (7.0%) | 0.52 |
| Malaria | Nil | Nil | - |

Data summarized as n (%) or mean (SD). SD: standard deviation.
